# The Influence of Autologous Bone Marrow Stem Cell Transplantation on Matrix Metalloproteinases in Patients Treated for Acute ST-Elevation Myocardial Infarction

**DOI:** 10.1155/2014/385901

**Published:** 2014-09-11

**Authors:** Eline Bredal Furenes, Trine Baur Opstad, Svein Solheim, Ketil Lunde, Harald Arnesen, Ingebjørg Seljeflot

**Affiliations:** ^1^Center for Clinical Heart Research, Department of Cardiology, Oslo University Hospital Ullevål, Postboks 4956, Nydalen, 0424 Oslo, Norway; ^2^Center for Heart Failure Research, Institute for Experimental Medical Research, Ullevål University Hospital, Kirkeveien 166, 0407 Oslo, Norway; ^3^Faculty of Medicine, University of Oslo, Postboks 1078, Blindern, 0316 Oslo, Norway; ^4^Department of Cardiology, Oslo University Hospital, National Hospital, Postboks 4950, Nydalen, 0424 Oslo, Norway

## Abstract

*Background*. Matrix metalloproteinase-9 (MMP-9), regulated by tissue inhibitor of metalloproteinase-9 (TIMP-1) and the extracellular matrix metalloproteinase inducer (EMMPRIN), contributes to plaque instability. Autologous stem cells from bone marrow (mBMC) treatment are suggested to reduce myocardial damage; however, limited data exists on the influence of mBMC on MMPs. *Aim*. We investigated the influence of mBMC on circulating levels of MMP-9, TIMP-1, and EMMPRIN at different time points in patients included in the randomized Autologous Stem-Cell Transplantation in Acute Myocardial Infarction (ASTAMI) trial (*n* = 100). Gene expression analyses were additionally performed. *Results*. After 2-3 weeks we observed a more pronounced increase in MMP-9 levels in the mBMC group, compared to controls (*P* = 0.030), whereas EMMPRIN levels were reduced from baseline to 2-3 weeks and 3 months in both groups (*P* < 0.0001). Gene expression of both MMP-9 and EMMPRIN was reduced from baseline to 3 months. MMP-9 and EMMPRIN were significantly correlated to myocardial injury (CK: *P* = 0.005 and *P* < 0.001, resp.) and infarct size (SPECT: *P* = 0.018 and *P* = 0.008, resp.). *Conclusion*. The results indicate that the regulation of metalloproteinases is important during AMI, however, limited influenced by mBMC.

## 1. Introduction

The early and late mortality after acute myocardial infarction (AMI) is declining, but cardiovascular disease (CVD) is still one of the leading causes of morbidity and death in the Western world. Ischemic heart disease is a feared, but often inevitable complication of atherosclerosis, the main underlying cause of myocardial infarction [1, 2]. Inflammation is considered to be a key process for development of atherosclerosis and this includes a number of cellular and molecular responses resulting in plaque formation [2, 3]. Despite well-documented treatment of AMI survivors, both medically and by percutaneous coronary intervention (PCI), some patients either do not receive this treatment, do not respond satisfactorily, or develop congestive heart failure despite treatment.

Several animal studies have shown that bone marrow stem cells differentiate to cardiomyocytes when infused into the affected myocardium [4]. Treatment with autologous stem cells from bone marrow has been suggested to reduce myocardial damage in patients with AMI. Results from clinical trials are, however, conflicting with regard to improvement of left ventricular ejection fraction [5–10]. Possible mechanisms by which autologous bone marrow stem cells act are discussed to be cardiac transdifferentiation, paracrine effects, angiogenesis, and reduced apoptosis [11, 12].

Matrix metalloproteinases (MMPs), a class of 24 endopeptidases, participate in plaque instability by degrading the extracellular matrix. MMP-9, a zinc-dependent gelatinase, is found in the shoulder of the plaque, contributes to plack instability and rupture, and has been associated with acute coronary syndrome (ACS) [3, 13]. Circulating MMP-9 has been shown to be elevated in patients with AMI, stable, and unstable angina pectoris [13, 14], as well as in hypertensives [15] and smokers [16]. In addition, MMP-9 is discussed to be involved in adverse left ventricular remodelling and associated with higher cardiovascular risk score [13]. The MMPs are regulated by specific endogenous inhibitors, tissue inhibitor of metalloproteinases (TIMPs), and MMP-9 is specifically regulated when pro-MMP-9 binds to TIMP-1 [14, 17].

Lately, the extracellular matrix metalloproteinase inducer (EMMPRIN, CD147), a member of the immunoglobulin superfamily, has been discussed to be involved in both expression and release of MMP-9 [18], thus, having a potentially regulatory role in CVD. EMMPRIN has been shown to be expressed in atherosclerotic plaques [19] as well as in cell types like monocytes, macrophages, and platelets [20].

In humans, limited data exists on the influence of bone marrow stem cells on MMPs that may be of importance for the myocardium and the infarction process. In the “randomized Autologous Stem-Cell Transplantation in Acute Myocardial Infarction” (ASTAMI) trial [10], the main aim was to assess the effects of intracoronary injection of autologous mononuclear bone marrow-derived cells (mBMC) on left ventricular ejection fraction in patients with ST-elevation myocardial infarction (STEMI). The main hypothesis was that the treatment would reduce the infarction sequelae, which, however, could not be demonstrated [10].

The aim of this ASTAMI substudy was to investigate the influence of coronary injection of mBMC on MMP-9, TIMP-1, and EMMPRIN, circulating levels as well as on gene expression in leukocytes, in patients with STEMI undergoing successful PCI. Furthermore, to investigate any association between the measured biomarkers and infarct size and left ventricular function, our hypothesis was that MMP-9 levels would be reduced after mBMC treatment, in parallel with reduction of EMMPRIN.

## 2. Materials and Methods

### 2.1. Subjects and Study Design

The ASTAMI study design and treatment protocol have previously been described in details [21]. Briefly, it was a randomized 1 : 1 open-labelled study, where one arm was intracoronary treatment with mBMC, and the other controls without aspiration and injection of bone marrow. Patients, age between 40 and 75 years, both gender were included. They were all treated with PCI with stent implantation in the left anterior descending (LAD) coronary artery.

Exclusion criteria were previous Q-wave infarction, cardiogenic shock, or severe comorbidity interfering with compliance to the protocol.

Baseline recordings were performed during day 4-5 after AMI, before bone marrow aspiration (in the treatment group) [10].

The study protocol, including the biobank, was approved by the Regional Committee for Medical Research Ethics and all patients gave written, informed consent. The study is registered at ClinicalTrials.gov, NCT 00199823.

### 2.2. Laboratory Methods

#### 2.2.1. Blood Sampling

A biobank, kept at −80°C consisting of plasma, serum, and PaxGene tubes (PreAnalytiX GmbH, Hombrechtikon, CH), the latter for gene expression measures in circulating leukocytes, was established. Blood samples were collected in fasting condition between 08.00 and 10.00 am the day before transplantation in the mBMC group (day-1) (baseline), the day after (day 1) and further day 3, after 2-3 weeks and after 3 months. The same time interval was used for the control group, except baseline sampling (day-1) which was drawn median 4 days after PCI compared to 5 days in the mBMC group.

#### 2.2.2. Enzyme Immunoassays

For analyses of MMP-9, TIMP-1, and EMMPRIN commercial enzyme linked immunosorbent assays (ELISA) (R&D Systems Europe, Abingdon, Oxford, UK) were used on serum samples, which were performed within 1 hour by centrifugation at room temperature 2500 ×g for 10 min. The interassay coefficients of variation (CV) were 7.3% for MMP-9, 4.4% for TIMP-1, and 5.4% for EMMPRIN.

#### 2.2.3. Gene Expression Analysis

Isolation of RNA from PaxGene tubes was performed according to the manufacturers instruction (PreAnalytix, Qiagen GmbH, Germany) in a subset of randomly selected samples (*n* = 47), with an additional cleaning step (Rneasy MinElute Cleanup Kit, Qiagen). A complementary DNA (cDNA) of the messenger RNA (mRNA) content was achieved by inversely transcribing total RNA in the samples. The genetic expression of mRNA of MMP-9 and EMMPRIN was performed by use of real-time PCR on the ViiA 7 Real Time PCR System (Applied Biosystems, Foster City, CA, USA) and the ΔΔCt method was applied [22]. This relative or comparative Ct method determines the relative target quantity (RQ-values) in the samples, by measuring the amplification (crossing threshold Ct) of the target samples and in a reference sample and normalized to an endogenous control (house-keeping gene). Assays for the target genes were Hs00234579_m1 for MMP-9, Hs00936295_m1 for EMMPRIN, and *β*-2 macroglobulin (Hs99999907_m1) was chosen as house-keeping gene.

#### 2.2.4. Measures of Myocardial Function

Left ventricular ejection fraction (LVEF) and infarct size were obtained by electrocardiogram-gated single photon emission computed tomography (SPECT) (GE Medical Systems with 4D-MSPECT software) at baseline (4.0 ± 1.4 days after the AMI). Infarct size was expressed as percentage of the LAD-area.

### 2.3. Statistical Analysis

MMP-9, TIMP-1, and EMMPRIN levels were all skewly distributed and nonparametric statistics were used throughout. Median values and 25 and 75 percentiles are given unless otherwise stated. For group comparisons the Mann-Whitney test was used for continuous data and Chi square test for categorical data. Friedman test was performed to analyze for differences between any time points within the groups. For changes from baseline (day-1) to the subsequent time points, Wilcoxon test was used only when Friedman test was significant. For differences in changes between the randomized groups, Mann-Whitney test was used. Spearman's rho was used for correlation analysis. The level of significance was set to *P* < 0.05. The SPSS software package version 18.0 was used throughout.

## 3. Results

Baseline characteristics of the study population according to the randomized groups are shown in Table 1.

One hundred patients included in the ASTAMI study, 50 randomized to mBMC treatment and 50 controls, were followed. From one patient we did not obtain blood samples. The groups did not differ regarding baseline characteristics. They were all medically treated according to current guidelines, thus, all patients were on statins, beta-blockers, ACE-inhibitors/ATII antagonists, and antithrombotic agents.

### 3.1. Circulating Levels of MMPs (Table 2)

Although blood samples at baseline were obtained one day later (median) from symptom start of AMI in the mBMC group compared to controls, no significant differences between the groups were recorded at baseline (day-1). No differences in MMP-9, TIMP-1, or EMMPRIN between the mBMC group and controls were seen at any further time points.

In the mBMC group there was a significant increase in MMP-9 levels from baseline to 2-3 weeks (*P* = 0.009), which could not be demonstrated in the control group. We observed no within group changes in TIMP-1 levels in either groups. EMMPRIN levels were significantly reduced from baseline to 2-3 weeks and 3 months in both groups (*P* < 0.0001, all).

When analyzing for differences between the groups in changes from baseline to further time points, we could demonstrate a significant difference in change in MMP-9 levels between the two groups at 2-3 weeks and after 3 months, showing a more pronounced increase in the mBMC group (*P* = 0.030 and *P* = 0.05, resp.). No differences in changes between the groups were observed for TIMP-1 and EMMPRIN.

### 3.2. Gene Expression Levels

**Figure 1:**
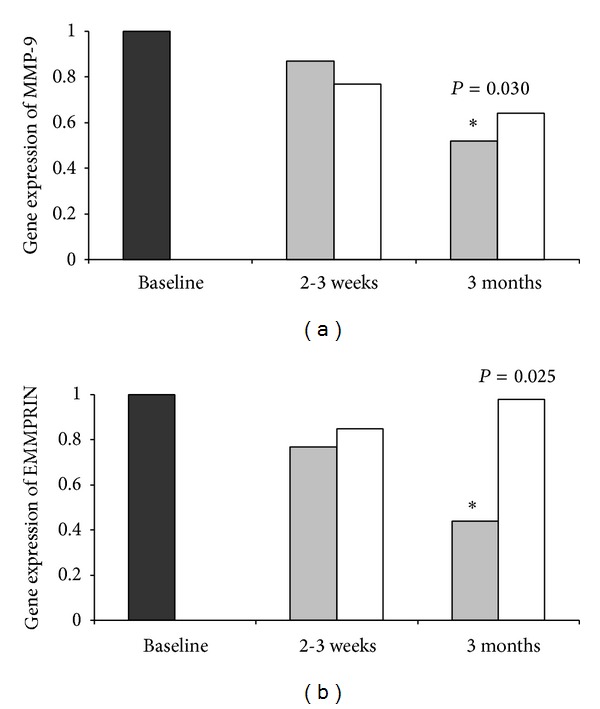
Gene expression of MMP-9 (a) and EMMPRIN (b) after 2-3 weeks and 3 months in the mBMC group and controls relative to baseline expression in the total population. Black bars: total group at baseline; grey bars: mBMC group; white bars: controls. ∗ indicates reduction from baseline to 3 months in the mBMC group *P* values refer to differences in expression relative to baseline between groups.

We observed no differences in MMP-9 gene expression between the two groups at any time points, while the EMMPRIN gene expression was significantly lower in the mBMC group (*n* = 23) versus the controls (*n* = 24) after 3 months (*P* = 0.03) (Table 3). The levels of both MMP-9 and EMMPRIN gene expressions were significantly reduced from baseline to 3 months in the mBMC group (*P* < 0.0001 and *P* = 0.002, resp.). This could not be demonstrated in the control group. There were, however, no differences between the groups in changes from baseline to any later time points. When defining baseline mRNA level (RQ-values) in the total population to 1, there was a 20% reduction in MMP-9 gene expression from baseline to 2-3 weeks, and a 50% reduction after 3 months in the mBMC group. A similar pattern was seen in gene expression of EMMPRIN, with a 20% reduction after 2-3 weeks and 60% reduction after 3 months in the mBMC group. The results of the gene expression presented as fold changes are illustrated in Figure 1.

### 3.3. Correlations

In the total population at baseline we observed a significant correlation between MMP-9 and EMMPRIN (*r* = 0.25, *P* = 0.011). A strong correlation was also shown between MMP-9 and TIMP-1 (*r* = 0.66, *P* < 0.0001) and also between TIMP-1 and EMMPRIN (*r* = 0.36, *P* = 0.01). Significant correlations between CK and baseline levels of both MMP-9 and EMMPRIN were found (*r* = 0.29  *P* = 0.005 and *r* = 0.43  *P* < 0.001, resp.). MMP-9 and EMMPRIN, but not TIMP-1, showed also significant correlations to infarct size measured by SPECT (*r* = 0.24, *P* = 0.018 and *r* = 0.27  *P* = 0.008, resp.). EMMPRIN levels were also found to be inversely correlated to LVEF at baseline (*r* = −0.31  *P* = 0.002).

We observed no significant correlations between circulating MMP-9 and gene expression of MMP-9 at any time points in the total population, or in the single groups. Likewise there were no correlations between circulating EMMPRIN and gene expression of EMMPRIN, or between gene expression of EMMPRIN and MMP-9 levels in either groups or in the total population.

## 4. Discussion

The main finding in the present study was that there was limited influence of intracoronary injection of mBMC transplantation after AMI on circulating levels of MMP-9, TIMP-1, and EMMPRIN, other than a more pronounced increase in MMP-9 after 2-3 weeks in the mBMC group. EMMPRIN levels were reduced after 2-3 weeks and 3 months in both groups. At baseline both MMP-9 and EMMPRIN were significantly correlated to myocardial injury assessed by biomarkers and infarct size and might therefore support their predictory ability for later outcome.

All patients were medically treated according to current guidelines; thus, any influence by medication on the measured variables would be equally affected in the randomized groups.

In both* in vitro* and* in vivo* studies, stem cell transplantation has been shown to reduce MMPs after AMI and improve ventricular remodeling [23]. Our hypothesis was therefore that treatment with mBMC would reduce the circulating levels of the selected biomarkers. In a study using modified mesenchymal stem cell transplantation into AMI rat hearts [24], a reduction in MMP-9 levels was shown. Mesenchymal stem cells are multipotent stromal cells that can differentiate into a variety of cell types [25], and the results are thus not quite comparable to ours. In addition, stem cell injection was performed one hour after AMI. In contrast, we found a significantly more pronounced increase in MMP-9 levels from baseline to 2-3 weeks in the mBMC group compared to controls. In accordance with our findings Roderfelt et al. demonstrated a transient inflammatory response and upregulation of MMP-9 activity after bone marrow transplantation in* Abcb*4^−/−^ (hepatic fibrosis) mice [26]. We have previously shown that MMP-9 levels are reduced 1 day after AMI [27]. Therefore we assume that the levels were normalized when baseline sampling in the present study was performed and limited influenced by the acute phase reaction. In the control group in our study, bone marrow aspiration was not performed. This procedure which itself is a trauma could influence the release of inflammatory markers and contribute to the elevated levels in the mBMC group.

In the study by Shu et al. using mesenchymal stem cell transplantation, TIMP-1 levels did not vary significantly [24], which is in accordance with our findings of no changes in this variable during the observation period in any of the groups.

The significant reduction in genetic expression of MMP-9 seen at 3 months might be discussed as compensatory to the increase observed in the circulating levels. MMP-9 expression is a crucial pathogenic feature in a range of conditions and disease states, also other than CVD [28–30], in which treatment with stem cells has been shown to suppress or downregulate the MMP-9 expression [30] and thereby improving the current condition.

The underlying mechanisms for the influence of stem cells on MMPs are not clarified. In cell culture of cardiac fibroblasts Wang et al. [31] could demonstrate that the protein expression and activity of MMP-2, but not MMP-9, were increased in response to hypoxia and decreased when cocultured with mesenchymal stem cells. It has also been demonstrated that early endothelial progenitor cells increased MMP-9 expression* in vitro*, whereas MMP-2 was increased in outgrowth endothelial cells [32]. The type of stem cells seems to be of importance regarding the degree of influence on MMPs [33].

The importance of EMMPRIN as an inducer of MMP-9 has been explored to a limited extent in humans. In our study circulating levels of MMP-9 and EMMPRIN were significantly correlated, indicating a common regulatory pathway [18]. Circulating levels as well as genetic expression of EMMPRIN were significantly reduced along with the increase in MMP-9. This might be discussed as a negative feedback mechanism. There was, however, no influence of mBMC on circulating levels or gene expression of EMMPRIN, shown by the significant reduction in both groups during the observation period. Expression of the EMMPRIN-gene in circulating leukocytes, also reported by Xu et al. assessed by flow cytometry [34], may indicate that the leukocytes contribute to the circulating levels, although no correlation between circulating levels and gene expression was observed. The reduction over time seen in EMMPRIN expression, with subsequent reduction in MMP-9 gene expression, contributes to the assumption that EMMPRIN is an inducer of MMP-9.

The significant correlations found between both MMP-9 and EMMPRIN, and myocardial injury assessed by biomarkers as well as infarct size measured by SPECT, have been sparsely explored in humans. In experimental AMI, MMP-9 has been shown to increase infarct size and left ventricular fibrosis [13], in accordance with our findings.

An association between EMMPRIN and the degree of myocardial injury and LVEF has previously been reported in the work by Nie et al. [35], but this was a post mortem immunohistochemistry study which showed a strong increase in EMMPRIN around the zone of necrosis in the AMI group. This can to some degree be compared to our findings of the correlations between MMPs and biomarkers of AMI and also to our results of an inverse correlation between EMMPRIN and LVEF. An additional contribution to this understanding has been demonstrated in CD147^+/−^ mice, where the disruption of the EMMPRIN-Cyclophilin A interaction reduced infarct size [36]. Our findings contribute to the suggestion that the expression of EMMPRIN is a decisive factor in regulating MMP-9 activity and thereby involved in myocardial remodeling.

The strength of the present study is the randomized design, the rather frequent sample collection for determination of the profiles, and gene expression analysis in a relatively large subpopulation. All patients were medically treated according to current guidelines; thus any influence by medication on the measured variables would therefore be equal in the randomized groups.

A limitation is that there was no bone marrow aspiration in the control group. There was, however, no significant difference between the two groups at baseline, that is, after the bone marrow aspiration in the mBMC group. It should be noted that the measures of circulating MMP-9 and TIMP-1 were performed in serum, as also used in many other studies. The proteins are released upon platelet activation during clotting, thus, the levels measured do not reflect the “true” circulating levels. However, the same standardized procedure for serum preparation was applied throughout the study, and therefore we assume that the comparisons between the randomized groups have been limited influenced. Confirmation of our results based on plasma samples is warranted. From the literature there are both coincide results and not when using plasma or serum samples, thus, it is important to take this into account when comparing results between studies. The gene expression analyses were performed in whole blood with circulating leukocytes as the RNA source, which might not be the most important source of either MMP-9 or EMMPRIN.

When performing stem cell transplantation as treatment regimen, several studies have discussed the timing, type of stem cells, and the procedure of the transplantation for optimization of the results [37, 38], but a conclusion has not yet been made.

## 5. Conclusions

Limited effects of intra coronary injection of mBMC transplantation on circulating levels as well as gene expression of MMP-9 and EMMPRIN in patients with STEMI treated with PCI could be demonstrated. EMMPRIN levels were reduced in both groups, whereas MMP-9 showed increased levels in the mBMC group. Both MMP-9 and EMMPRIN were significantly correlated to myocardial injury and infarct size, indicating that the regulation of metalloproteinases is important in the process of an AMI. The results contribute to the understanding of the pathophysiology of metalloproteinases in AMI, but further investigations are needed regarding timing and type of stem cells.

## Figures and Tables

**Table 1:** Characteristics of the study population according to the randomized groups. Values are presented as proportion, means ± SD, or medians^1^ with 25th and 75th percentiles.

	mBMC group (*n* = 50)	Control group (*n* = 50)
Age (years)	58.1 (8.6)	56.7 (9.6)
Gender (% female)	16	16
Hypertension (%)	35	34
Diabetes (%)	10	8
Smokers (%)	39	48
BMI (kg/m^2^)	26.3 (3.3)	27.1 (4.1)
SBP/DBP (mmHg)	131/82 ± 21/14	132/83 ± 23/17
Tot. chol (mmol/L)	4.4 ± 0.9	4.5 ± 0.9
LDL chol (mmol/L)	2.9 ± 0.8	2.9 ± 0.7
HDL chol (mmol/L)	1.0 ± 0.3	1.1 ± 0.3
Triglycerides (mmol/L)^1^	1.3 (1.0, 1.7)	1.3 ± (1.1, 1.5)
Symptom start to PCI (min)^1^	210 (180, 330)	230 (180, 330)
LVEF (%)	41.3 (10.6)	42.6 (11.7)
Infarct size (%)	43.7 ± 17.6	40.7 ± 19.3
Peak CK (U/L)	3338 ± 2398	3532 ± 2650
Thrombolysis before PCI (%)	30	28
Medication at discharge		
Aspirin (%)	100	100
Clopidogrel (%)	100	100
ACE-I/ATII antagonist (%)	100	100
*β*-blocker (%)	100	100
Diuretics (%)	43	32
Statin (%)	100	100

ATII antagonist: angiotensin II receptor antagonist; ACE-I: angiotensin-converting enzyme inhibitor; BMI: body mass index; DBP: diastolic blood pressure; LVEF: left ventricular ejection fraction; PCI: percutaneous coronary intervention; and SBP: systolic blood pressure.

**Table 2:** Circulating levels of MMP-9, TIMP-1, and EMMPRIN at baseline and follow-up in the randomized groups. Median values (25th and 75th percentiles) are given.

	Baseline (Day-1)		Day 1		Day 3		2-3 weeks		3 months	
	mBMC	Control	mBMC	Control	mBMC	Control	mBMC	Control	mBMC	Control
MMP-9 (ng/mL)	205 (79, 419)	235 (83, 502)	286 (98, 432)	276 (103, 518)	287 (115, 426)	273 (116, 499)	**281** ^∗†^ (141, 574)	302 (106, 419)	**270** ^†^ (153, 410)	210 (110, 398)

TIMP-1 (ng/mL)	211 (158, 326)	228 (151, 327)	234 (157, 326)	253 (158, 354)	222 (173, 322)	253 (182, 333)	216 (171, 336)	213 (172, 306)	204 (164, 282)	220 (182, 273)

EMMPRIN (pg/mL)	4324 (3663, 4878)	4329 (3752, 4994)	4317 (3739, 4966)	4357 (3976, 4952)	4339 (3667, 4837)	4367 (3768, 4853)	**3754** ∗ (3249, 4436)	**3857** ∗ (3440, 4476)	**3654** ∗ (3312, 4099)	**3775** ∗ (3364, 4245)

No differences between the groups were observed at any time points.

∗Refers to *P* value < 0.05 for intragroup changes from baseline to later time points.

^†^Refers to *P* value ≤ 0.05 for differences in changes from baseline between the groups.

**Table 3:** The levels of gene expression, RQ values of MMP-9, and EMMPRIN at baseline and follow-up.

	Baseline (Day-1)		Day 1		Day 3		2-3 weeks		3 months	
	mBMC	Control	mBMC	Control	mBMC	Control	mBMC	Control	mBMC	Control
MMP-9	0.483 (0.309, 0.749)	0.441 (0.289, 0.826)	0.460 (0.255, 0.772)	0.466 (0.322, 0.655)	0.384 (0.229, 0.652)	0.379 (0.265, 0.543)	0.444 (0.328, 0.760)	0.395 (0.275, 0.640)	**0.247** ∗ (0.159, 0.367)	0.357 (0.223, 0.521)

EMMPRIN	0.567 (0.248, 0.961)	0.566 (0.305, 1.016)	0.358 (0.251, 0.797)	0.467 (0.226, 0.953)	0.490 (0.196, 0.758)	0.273 (0.174, 1.005)	0.512 (0.231, 0.810)	0.567 (0.182, 0.927)	**0.295** ^∗#^ (0.177, 0.533)	0.649 (0.223, 1.166)

*Refers to *P* value < 0.05 for intragroup changes from baseline to later time points.

^#^Refers to *P* value < 0.05 for differences between the groups.
